# Multidisciplinary work promotes preventive medicine and health education in primary care: a cross-sectional survey

**DOI:** 10.1186/s13584-019-0318-4

**Published:** 2019-06-06

**Authors:** Ayelet Schor, Lucia Bergovoy-Yellin, Daniel Landsberger, Tania Kolobov, Orna Baron-Epel

**Affiliations:** 1 School of Public Health, University of Haifa, Haifa, Israel; 2 Sde-varburg, Israel; 3 Department of Health Services Research and Health Economics, Maccabi Healthcare Services, 27 Ha’Mered Street, Tel Aviv, Israel; 4 Maccabi Healthcare Services, 3 Ha’Netsach Street, Ramat-Hasharon, Israel; 5 School of Education, Bar-Ilan University, Ramat Gan, Israel; 6 Qyriat Bialik, Israel; 7 School of Public Health, University of Haifa, 199 Abba khoushy Mount Carmel, 3498838 Haifa, Israel

**Keywords:** Primary care model, Preventive medicine measures, Health education tools, Multidisciplinary practice

## Abstract

**Background:**

Preventive medicine and health education are among the strategies used in coping with chronic diseases. However, it is yet to be determined what effect do personal and organizational aspects have on its’ implementation in primary care.

**Methods:**

A cross-sectional survey was conducted in order to assess and compare preventive medicine and health education activities in three types of primary care models: solo working independent physicians, nurse-physician collaborations and teamwork (nurses, dietitians and social workers working alongside a physician). Questionnaires were emailed to 1203 health professionals between September and November 2015, working at Maccabi Healthcare Services, the second largest Israeli healthcare organization.

Self-reported rates of health education groups conducted, proactive appointments scheduling and self-empowerment techniques use during routine appointments, were compared among the three models. Independent variables included clinic size as well as health professionals’ occupation, health behaviors and training.

A series of multivariate linear regressions were performed in order to identify predictors of preventive medicine and health education implementation.

Computerized health records (CHR) validated our self-report data through data regarding patients’ health behaviours and outcomes, including health education group registration, adherence to occult blood tests and influenza vaccinations as well as blood lipid levels.

**Results:**

Responders included physicians, nurses, dietitians and social workers working at 921 clinics (*n* = 516, response rate = 31%).

Higher rates of proactive appointments scheduling and health education groups were found in the Teamwork and Collaboration models, compared to the Independent Physician Model. Occupation (nurses and dietitians), group facilitation training and personal screening adherence were identified as preventive medicine and health education implementation predictors.

Group registration, occult blood tests, healthy population’s well-controlled blood lipids as well as influenza vaccinations among chronically ill patients were all significantly higher in the Teamwork and Collaboration models, compared to the Independent Physician Model.

**Conclusions:**

The Teamwork and Collaboration models presented higher rates of preventive medicine and health education implementation as well as higher rates of patients’ positive health behaviours documented in these models.

This suggests multidisciplinary primary care models may contribute to population’s health by enhancing preventive medicine and health education implementation alongside health professionals’ characteristics.

## Background

Non-adherence to medical and behavioral recommendations is common and is known to be affected by patients and physicians’ characteristics alike [1–4].

Recent studies indicate that health professionals have the ability to improve patients’ adherence using various behavioral tools and strategies for change [5–7].

One of the main strategies found to improve treatment processes and access to medical care, resulting in improved clinical outcomes [8–10] is multidisciplinary work. It is assumed that multidisciplinary collaborations increase the ability to accurately address a patient’s individual needs, resulting in better adherence to treatment [11].

In order to achieve these goals, health professionals engage in health education in an attempt to educate and activate their patients. Health educations is defined as creating learning opportunities designed to allow patients to accept informed decisions and to promote positive health behaviors that would improve their health [12].

Health education utilizes a variety of tools designed to enhance patients’ motivation and adherence.

One of these tools is the use of health education groups, where trained health professionals serve as group facilitators, guiding the participants in the acquisition of practical tools that promote desired behavioral changes.

These groups seem to be an effective preventive medicine tool, as participation helps in a variety of behavioral challenges such as weight loss, smoking cessation, and self-management of chronic illnesses [13, 14].

Another health education tool is the use of proactive appointments, initiated by the health provider rather than the patient. This allows health professionals the opportunity to focus on preventive medicine counseling, perform routine check-ups and use empowerment tools, such as motivational interviewing [15–17], in order to facilitate patients’ adherence.

In spite of abundant supportive evidence indicating that the use of preventive medicine and health education tools reduces morbidity and mortality [18, 19], its’ implementation may be complicated and ultimately depends on health professionals’ motivation, affected by multi-level inter-personal, and organizational factors [20].

This study examined three primary care models implemented by Maccabi Healthcare Services (MHS). MHS is the second largest health maintenance organization (HMO) in Israel, with over two million clients, representing about a quarter of the country’s population [21].

The basic primary care model, the Independent Physician Model, was developed when MHS was founded in 1940 [22]. Independent physicians work solo in private clinics. They are encouraged to achieve MHS’ desired clinical outcomes, such as patients’ vaccinations, and their income (per capita) is supplemented accordingly.

The second model examined is the Teamwork Model, based on the Chronic Care Model [23, 24] first implemented in MHS in 2005. Teamwork clinics employ various health professionals (physicians, nurses, dietitians and social workers). Composition of the teams vary among clinics, some include all four professions and others only two (a physician and one other health profession). The type of health professions, as well as the amount of weekly / monthly hours assigned to the team varies according to the population’s needs as well as MHS’ ability to supply specific demands. Thus, some clinics are based mostly on a physician and nurse with a few weekly / monthly hours of a dietitian and social worker, while others revolve around a physician and dietitian with a few nursing hours a week. Regardless of team composition, MHS expects all Teamwork clinics to apply multi-disciplinary work strategies, such as regular staff meetings, conducted in order to discuss patients’ treatment. Teamwork strategies, however, are not monitored by MHS as a part of clinics’ assessment conducted on a regular basis, so there is no objective data as to how common teamwork practices really are.

All Teamwork health professionals receive a monthly salary, independent of patients’ outcomes, with the exception of physicians, who enjoy additional financial incentives similar to Independent physicians. As teamwork clinics are expected to focus on preventive medicine and patient self-management, health professionals affiliated with these clinics are prioritized when resources are allocated, for example when relevant training take place.

Attempting to provide different solutions to different needs and limited resources, in 2013, MHS began implementing the Collaboration Model. This Model stems from the Independent Physician Model and follows its financial model. Independent nurses collaborate with one to four adjacent primary care independent physicians who refer patients to their affiliated nurse when they see the need for a nurse’s intervention, such as blood pressure monitoring, diabetes counseling, health education on other issues etc.On the other hand, when the independent nurses require consultations or see the need for treatment by physicians (like medication changes), they will refer the patient to their affiliated physician.

Patients choose their primary care physician and are mostly unaware of their affiliation to a specific primary care model. Accordingly, distributions of patients’ main characteristics such as gender, age and morbidity levels, defined by Charlson score [25, 26] is mostly similar among the models. Most patients, in all models are males, Collaboration patients are a little younger with higher morbidity levels (significance mostly stemming from the large sample size).

Table 1 presents the organizational and patients’ characteristics among the three primary care models.

**Table 1:** Organizational and Patients’ Characteristics among the Three Primary Care Models^1,2^

	Independent physician model	Team model	Collaboration model
	*n* = 92	*n* = 264	*n* = 30
Personnel	Physicians	Physicians	Physicians
		Nurses	
		dietitians	Nurses
		Social-worker	
Clinic Ownership	Physician’s clinic	MHS’ clinic	Physician’s clinic
			Nurse’s clinic
Supporting staff	Physicians’ choice	MHS’ allocation	Physicians’ / Nurses’ choice
Patients’ Affiliation	Physician	Physician	Physician
			Nurse
Clinical Goals	Physicians’ goals	Physicians’ goals	Physicians’ goals
		No team goals	Nurses’ goals
Fee Policies	Per capita Supplementation according to defined outcomes achieved	Physicians Only	Physicians and Nurses
		Per capita	Per capita
		Supplementation according to defined outcomes achieved	Supplementation according to defined outcomes achieved
		All other professional	Nurses only
		Salary	Supplementation according to defined counseling activities conducted (smoking cessation counseling, diabetic guidance etc.)
		No supplementation according to defined outcomes achieved	
Patients’ Gender (Male)^1,2^	55.8	52.3	58.4
Patients’ Age (Years) ^2,3^	64.3 ± 12	64.2 ± 12	63.4 ± 13
Patients’ Morbidity Levels ^2,3,4^	1.04 ± 1.26	1.08 ± 1.27	1.10 ± 1.25

^1^Percentage within the model’s population

^2^*p* < 0.001

^3^Mean ± SD

^4^Charlson Score on December 31st, 2015

All health professionals (physicians, nurses, dietitians and social workers), regardless of the model they are affiliated with, are encouraged to acquire preventive medicine skills and implement relevant activities in their clinics. For that reason training is conducted within working hours and if not, health professionals are reimbursed for their time and training costs. In addition, professionals gain training points upon completion, granting them with additional income. Trained health professionals are free to conduct health education group counseling in all MHS’ clinics and their income is supplemented accordingly.

Health education groups are available for all patients in multiple communal MHS facilities, regardless of the clinical model they belong to. This allows clinics that do not include trained health professionals but want to pursuit preventive medicine, to refer their patients to health education groups conducted in a nearby clinic, or to conduct one in their own clinic, guided by trained MHS health professionals, not affiliated with their own clinic.

Regretfully, while the benefits of preventative medicine and health education tools have been previously established [18, 27, 28], in reality, it is applied sporadically.

Moreover, its’ implementation is not taken into account in routine clinic assessments, as other key components, such as patients’ medication adherence or hospitalizations are.

Acknowledging that certain organizational aspects may affect the implementation of preventive medicine and health education tools, this study aimed to explore the use of such tools in various primary care models.

Our aim was to better understand what part do personal and organizational aspects play in the implementation of preventive medicine tools within the different primary care models implemented by MHS.

Since preventive medicine is highly encouraged by MHS in the Teamwork clinics, we assumed health professionals affiliated with the Teamwork Model would apply preventative medicine and health education tools more widely.

## Methods

A cross sectional survey was conducted between September and November 2015.

Questionnaires were emailed during 2015 to all health professionals (1203 physicians, nurses, dietitians, and social workers) affiliated with one of the clinics included in this study, all of whom had been employed in the clinic for at least 12 months prior to the data collection date. All clinics who were operational for at least 12 months prior to the data collection date were included in our study. Respondents included 92 physicians from 594 Independent Physician clinics, 30 professionals from 52 Collaboration clinics, and 264 members of 273 Teamwork clinics, with 16, 78 and 43% response rates respectively, overall response rate of 31%. (Distribution of clinic and health professionals’ personal characteristics by primary care model affiliation is presented in Table 2).

**Table 2:** Distribution of Clinic and Health Professionals’ Personal Characteristics by Primary Care Model Affiliation^a, b^

Variable	Independent physician model	Team model	Collaboration model	Value^b^	*p*-value
	*n* = 92	*n* = 264	*n* = 30		
Clinic size (No. of patients) (%)					
Up to 400	10.6	6.2	3.3	18.35	0.001
400–1000	38.8	19.1	20		
0ver 1000	50.6	74.7	76.7		
Personal characteristics (%)					
Occupation					
Physician	100	28.8	40	152.86	*p* < 0.001
Nurse	0	36.9	60		
Dietitian	0	20.7	0		
Social worker	0	13.4	0		
Gender					
Male	56.2	15.7	32.1	47.68	*p* < 0.001
Female	43.8	84.3	67.9		
Participation in patient empowerment training					
Yes	15.7	52.1	51.7	26.34	*p* < 0.001
No	84.3	47.9	48.3		
Health behaviors (%)					
Smoking					
Yes	3.8	2.9	10.7	4.03	0.13
No	96.2	97.1	89.3		
Undergoing regular screening					
Yes	75.0	84.3	78.6	3.46	0.17
No	25.0	15.7	21.4		
Regular physical activity					
Yes	73.7	61.8	41.2	4.31	0.11
No	26.3	38.2	57.1		

^a^Percent

^b^Chi-square/Fisher Test

Questions were presented in Hebrew (translated to English by the authors for publication purposes). The questionnaires were completed anonymously via an automated system, preventing respondents’ identification. Five weekly automatic reminders were sent to those who did not open the questionnaire link.

Data was extracted, processed and analyzed by MHS’s Department of Health Services Research.

Respondents fully represented the health professionals affiliated with the clinic examined, with respect to gender, clinic size, and occupation, as presented in Table 3. Physicians and small clinics were under-represented, partly due to the fact that some health professionals, mainly physicians were affiliated with more than one clinic. While small clinics may differ in their ability to implement preventative medicine tools, as mentioned, MHS activities are available to all patients, regardless of the clinic they are affiliated with.

**Table 3:** Respondents versus MHS’ health professionals affiliated with the three Primary Care Models

			Gender		Clinic size		
			Male	Female	Small	Medium	Large
Independent Physician Model		Percentage among respondents	57	43	9.8	37	53.3
		Percentage among MHS’ employees	62.6	37.4	16.3	19.9	53.8
Team model	Physicians	Percentage among respondents	55.6	44.4	1.3	26.7	72
		Percentage among MHS’ employees	56.2	43.8	57.1	26.2	17
	Nurses	Percentage among respondents	4.7	95.3	1.1	5.4	93.5
		Percentage among MHS’ employees	2.8	96.2	3.6	47.8	60.7
	Dietitian	Percentage among respondents	2.2	97.8	24	34	42
		Percentage among MHS’ employees	3.7	96.3	16.7	28.7	54.6
	Social Worker	Percentage among respondents	16.7	83.3	3.4	13.8	82.8
		Percentage among MHS’ employees	19.3	80.7	4.2	11.4	84.3
Collaboration Model		Percentage among respondents	11.8	88.2	5.6	27.8	66.7
		Percentage among MHS’ employees	5.2	94.8	24.2	29.5	46.3

Regretfully, a low number of dietitians took part in the study. Since dietitians’ work characteristics are more similar to those of nurses than to social workers, they were added to the nurses for analysis.

The *dependent variables* included three aspects representing the application of preventative medicine and health education tools in the clinic.

Health education groups implementation was examined by the reported frequency of running these groups in the clinic. Acknowledging the challenges of organizing health education groups, our aim here was to evaluate to what extent do health professionals implement this type of intervention in their clinics, regardless of the type of group conducted.

Therefore, the question was: “How often are smoking cessation groups / diabetes groups / group educational events, conducted in the clinic?” Responses ranged from 1 = never to 4 = 3 a year or more.

Proactive medicine was evaluated by the frequency of proactive appointments (a common organizational term) scheduled, reported by responses to the question: “How often do you schedule proactive appointments for your patients?” Responses ranged from 1 = never to 4 = regularly.

The routine use of patient empowerment techniques was examined by the question: “How often do you use empowerment techniques during routine appointments?” Responses ranged from 1 = never to 4 = in most appointments.

Since these variables consisted of four categories and the answers did not distribute normally, they were dichotomized by combining the three lower scores into “low frequency to none”, and “high frequency” represented by the highest score.

These three dependent variables were based on health professionals’ self-report, as there is no existing objective quantitative data regarding these variables.

### Computerized data

In order to support the self-reported data, we added computerized data extracted from MHS’ computerized health records (CHR) to our analysis.

We extracted data regarding 2015 prevalence rates of chronically ill patients in each primary care model as documented in MHS’ chronic illness automatic registration system [29, 30], as well as registration rates to health education groups. This enabled us to objectively assess rates of registration to group counseling conducted.

We also examined a few health behavior outcomes documented in the CHR. This helped to gain a broader prospective and to assess whether activities implemented may indeed be associated with enhanced health outcomes. This data included the whole cohort of patients in Maccabi (Independent Physician Model *n* = 464,828, Team Model *n* = 269,844 and Collaboration Model *n* = 60,778).

We chose health outcomes that are included in the Israeli quality indicators program, representing primary, secondary and tertiary prevention and may be improved with the support of trained health professionals. Among the healthy population (MHS members not in one or more of the chronically ill registries), we examined rates of conducting occult blood tests and well-controlled lipid levels. Influenza vaccination rates among diabetic patients as well as heart diseases and high blood-pressure patients were also examined. (Yes / No for all variables). These health outcomes are defined by the Israeli Quality Health Indicators Plan, shown to significantly affect various health behaviors as well as chronically ill patients’ health status [12, 31].

The *independent variables* consisted of the respondents’ personal characteristics as well as clinic characteristics.

### Clinic characteristics

Included the type of model (Independent Physician / Teamwork / Collaboration) and clinic size (small< 400 patients, medium = 400–1000 patients, large> 1000 patients).

### Personal characteristics

Gender, occupation and personal health behaviors (smoking, regular physical activity, and adhering to relevant health screening).

### Training

Professional’s participation in health education training (motivational interviewing / smoking cessation counseling / group facilitation), as well as time passed since completion of this training (1–6 months, 7–12 months, 13–24 months, over 24 months).

Training data was analyzed as an ordinal variable. Each type of training was given a value reflecting the time elapsed since completion (1 = no training, 2 = 1–6 months, 3 = 7–24 month, 4 = over 24 months).

## Statistical analysis

The data were analyzed using the statistical program for Health and Welfare Science for Windows (SPSS, version 22.0, Chicago, IL, USA). Descriptive data analysis included range, mean and standard deviation for continuous variables, and frequency and percentage for categorical variables. Personal characteristics were analyzed using Chi-square and Fisher tests.

Comparisons of means regarding health education application among the three models were performed using Kruskal-Wallis tests.

Between groups comparisons were conducted in order to assess between which two groups differences were significant. Bonferroni for distribution of clinic and health professionals’ characteristics as well as for preventive medicine implementation and Pairwise contrasts for patients’ health results.

Logistic regression models were conducted in order to identify predictors of health education implementation.

The significance for all the statistical tests was set to 0.05.

## Results

The clinic and personal characteristics are presented in Table 2.

The majority of respondents affiliated with the Teamwork and Collaboration models were women, while in the Independent Physician Model most were men (*p* < 0.001).

As a whole, the health professionals reported high rates of positive health behaviors, and the differences among the models were not statistically significant.

Reported rates of health education training were similar in the Teamwork and the Collaboration models (about 52%), as opposed to only 16% in the Independent Physician Model (*p* < 0.001).

Table 4 presents the average scores of implementing preventative medicine and health education tools in the three models.

**Table 4:** Average Score of Preventive Medicine Tools Implementation among the Models^a, b, c^

	Independent Physician model *n* = 92			Teamwork model *n* = 264			Collaboration model *n* = 30				
	mean	C.I 95%		mean	C.I 95%		mean	C.I 95%		F	Sig.
		Low.	Up.		Low.	Up.		Low.	Up.		
Conduct group counseling activities (1 = never to 4 = three a year or more)	1.7	1.44	1.89	3.2	3.02	3.28	3.1	2.71	3.50	66.844	*p* < 0.001
Schedule proactive appointments (1 = never to 4 = regularly)	3.0	2.82	3.25	3.7	3.62	3.78	3.8	3.56	3.95	27.687	*p* < 0.001
Use of empowerment techniques during routine appointments (1 = never to 4 = in most appointments)	3.0	2.81	3.23	3.2	3.09	3.30	3.3	3.02	3.60	1.773	0.17

^a^Data are presented as mean scores± standard deviation

^b^ANOVA test

^c^1 = lowest frequency, 5 = highest frequency

Overall, respondents from the multidisciplinary models (Collaboration and Teamwork models) reported similarly higher rates of proactive medicine and health education tools implementation compared with the Independent Physician Model.

The multidisciplinary models reported significantly higher rates of health education group counseling conducted in the clinics (mean scores of 3.2, 3.1 and 1.7 for the Teamwork, Collaboration and Independent Physician models respectively *p* < 0.001).

Registration to health education group counseling among chronically ill patients was higher in the multidisciplinary models. Registration rates ranged from 2.19% in the Independent Physician Model (*n* = 4596/209,385), through 2.29% (*n* = 3436/149,412) in the Teamwork Model, to 3.05% (*n* = 764/24,995) in the Collaboration Model (*p* < 0.001).

Rates of proactive appointment scheduling in the clinic were also significantly higher in the multidisciplinary models compared with the Independent Physician Model (mean score of 3.8 and 3.7 and 3.0 for the Collaboration, Teamwork and Independent Physician models respectively, *p* < 0.001).

No significant differences were observed between the models regarding the use of empowerment techniques (*p* = 0.17). However, they were higher in the multidisciplinary models.

Variables that may predict the implementation of proactive medicine tools were identified through multivariable linear regression models.

Table 5 presents predictors of preventative medicine and health education tools implementation.

**Table 5:** Logistic Regression Analysis Presenting Predictors of Preventative Medicine Implementation in the Clinics ^a, b^

Variable	Category	OR	95% C.I.		Sig.
			Lower	Upper	
Conducting health education group counseling in the clinic					
Primary care model type	Independent Physician	1.000			
	Collaboration	4.33	1.38	13.57	0.01
	Teamwork	6.09	2.63	14.13	*p* < 0.001
Occupation	Physician	1.000			
	Nurse + Dietitians	2.08	1.09	3.95	0.02
Training	Group facilitation	0.77	0.36	1.63	0.50
	Smoking cessation	2.09	0.79	5.52	0.13
Health behaviors	Adhering to regular screening	1.18	0.59	2.35	0.63
Scheduling proactive appointments					
Primary care model type	Independent Physician	1.000			
	Collaboration	0.77	0.21	2.74	0.68
	Teamwork	2.09	1.04	4.19	0.03
Occupation	Physician	1.000			
	Nurse + Dietitians	28.46	8.58	94.42	*p* < 0.001
Training	Group facilitation	1.52	0.58	3.94	0.38
	Smoking cessation	3.95	1.15	13.53	0.02
Health behaviors	Adhering to regular screening	0.95	0.47	1.93	0.90
Using patient empowerment techniques in routine appointments					
Primary care model type	Independent Physician	1.000			
	Collaboration	0.63	0.21	1.81	0.39
	Teamwork	0.53	0.26	1.07	0.08
Occupation	Physician	1.000			
	Nurse + Dietitians	1.77	0.92	3.42	0.08
Training	Group facilitation	2.76	1.29	5.92	0.009
	Smoking cessation	0.79	0.30	2.10	0.64
Health behaviors	Adhering to regular screening	2.29	1.14	4.59	0.01

^a^The table presents only significant predictors

^b^Variables included in the linear regression and were not significant: clinic size, gender, social worker occupation, motivational interview training, regular physical activity, smoking

The type of primary care model significantly predicted health education groups counseling conducted in the clinic. Respondents from the Teamwork Model and the Collaboration Model were much more likely to conduct group counseling activities in the clinics than those of the independent physician model (OR = 6.1, 95%CI 2.63–14.13 and OR = 4.3, 95%CI 1.38–13.57 respectively). The Teamwork Model was not significantly different from the Collaboration Model and both were significantly different from the Independent physician Model (*p* < 0.001).

Another significant predictor of group-counseling activities was the type of occupation. Nurses and dietitians were twice as likely to conduct group counseling, compared to physicians (OR = 2.08, 95%CI 1.09–3.95).

Significant predictors of proactive appointment scheduling included affiliation with the Teamwork Model (OR = 2.1, 95%CI 1.04–4.19), occupation, namely nurses and dietitians (OR = 28.46, 95%CI 8.58–94.4) and training, specifically smoking cessation training (OR = 3.95, 95%CI 1.15–13.53). The Teamwork Model was not significantly different from the Collaboration Model and both were significantly different from the Independent physician Model (*p* < 0.001).

Significant predictors for the use of patient empowerment techniques during routine appointments included group facilitation training and health professionals’ adherence to regular screening (OR = 2.77, 95%CI 1.30–5.92; OR = 2.29, 95%CI 1.14–4.49 respectively).

Rates of most patients’ health outcomes we examined were significantly higher in the Teamwork and Collaboration models (presented in Table 6).

**Table 6:** Rates of Health Behaviours among the Three Primary Care Models^a^

Variables	Independent physician model	Team model	Collaboration model	Value^b^	*p*-value
Healthy Population					
	*n* = 464,828	*n* = 269,844	*n* = 60,778		
Occult Blood Test	60.0	61.6	61.4	90.51	*p* < 0.001
Well-Controlled Lipid Levels^c^	91.1	91.6	91.5	210.11	*p* < 0.001
Diabetic Population					
	*n* = 51,861	*n* = 37,455	*n* = 6006		
Influenza Vaccination	55.2	56.0	60.4	58.04	*p* < 0.001
Heart Diseases Population					
	*n* = 37,036	*n* = 25,239	*n* = 4509		
Influenza Vaccination	57.9	59.1	63.3	49.71	*p* < 0.001
High Blood-Pressure Population					
	*n* = 120,349	*n* = 86,629	*n* = 14,461		
Influenza Vaccination	46.0	46.1	50.2	96.19	*p* < 0.001

^a^Percent among target population

^b^ Chi-square

^c^ Among patients who underwent lipid levels check-ups during 2015

The only variable in which the Independent Physician Model presented similar results to those of the Teamwork Model was the influenza vaccination among diabetic patients (46.01 and 46.2% respectively). However, the Collaboration model presented significantly higher rates of diabetic influenza vaccination compared with those of the Independent Physician Model (*p* < 0.018).

## Discussion

This study aimed at identifying factors that support or hinder the implementation of preventive medicine and health education tools in various primary care models.

Combining health professionals’ self-report with computerized objective data enabled us to gain insights regarding personal and organizational aspects that are associated with the implementation of preventive medicine and health education tools. While this study examined MHS, our findings are likely to be relevant to other Israeli HMOes, as well as other countries implementing similar primary care models.

Our results found that the health professionals affiliated with the multidisciplinary models engaged in more training and implementation of preventive medicine and health education tools compared to those affiliated with the Independent Physician Model. More specifically, significantly higher rates of proactive appointments scheduling and health education groups were found in the Teamwork and Collaboration models, compared to the Independent Physician Model.

This confirmed our basic hypothesis that Teamwork enhances the implementation of preventive medicine and supports previous research that demonstrated Teamwork to yield better patient adherence as well as improved clinical outcomes [10].

To our surprise, while the Collaboration Model and the Independent Physician Model operate under similar organizational approaches, results presented by the Collaboration Model resembled those of the Teamwork Model.

The significant predictors of preventive medicine implementation we identified can be divided into two mutually interactive levels: the personal level (health professionals) and the organizational level (MHS). These multi-leveled interactions were previously established by DiClemente et.al. as influencing behavioral choices among employees, in our case, their choice to implement preventive medicine tools [20].

The significant *Personal level factors* we identified included health behaviors (namely adhering to regular health screening), training and occupation, all identified as relevant in previous studies [3, 4, 15]. We found nurses were 28.5 more times likely to schedule proactive appointments. This is in line with previous studies demonstrating the significance of the nursing profession in proactive medicine within primary care [17]. Moreover, preventative medicine is traditionally conducted primarily by nurses in Israeli practices. This is well described in a recent Israeli survey that found physicians perceiving nurses as contributing to practice quality and as sharing the responsibility for quality of care [32].

The *organizational level factors* demonstrated the significance of the type of primary care model manifested by different combinations of health professionals affiliated with the clinics, as well as different policies such as resources allocated or fee, varied among the models and the professions. The significance of these aspects is addressed later.

Relevant training was highly effective as we did find that primary care models with a higher percentage of trained health professionals implemented more preventive medicine and health education tools. Moreover, the more experienced the professionals were in group facilitation, the more likely they were to use empowering techniques in their routine appointments.

On the other hand, high rates of proactive appointment scheduling and health education group registration in the Independent Physicians’ Model were seemingly contradictory to the low training levels reported by this model’s respondents.

This may be attributed to the fact that physicians affiliated with this model do not have nurses to depend upon in encouraging patients to participate in relevant activities, as commonly done in Israeli multidisciplinary teams [32], prompting them to do so on their own.

The use of empowerment techniques during routine appointments may be influenced by personal level factors, such as positive attitudes towards patient empowerment, while organizational factors such as clinic space do not play a role in the use of this tool. This may explain why we found the three models did not differ where the use of empowerment techniques in routine appointments was concerned.

Various fee policies implemented by MHS may have also influenced the decision to apply preventive medicine tools. However, while financial incentives have been found to be effective in improving processes of care and achieving targeted outcomes [33] our results challenge this perception.

The Independent Physician and the Collaboration models, both enjoying financial incentives, presented significantly different results regarding preventive medicine. Furthermore, in the Teamwork model, respondents reported high rates of preventive medicine and health education tools implementation despite the lack of incentives for health professionals, other than the physicians. Moreover, Teamwork nurses and dietitians were strongly associated with higher levels of group counseling and proactive appointment scheduling. This implies that financial incentives may not necessarily promote the implementation of preventive medicine tools and its’ specific effect require further investigation.

Result concerning preventive medicine implementation as well as patients’ health results, may be associated with the different sample sizes among the primary care models. In between two groups differences regarding patients’ health outcomes may be attributed to large sample sizes. Never the less, the fact that Collaboration Model presented similar results to that of the Teamwork Model, which requires considerable organizational investment, raises the need to reassess organizational policies regarding these models. Fee policies, personnel allocation etc. should be re-evaluated in order better utilize existing resources while maintaining optimal patients’ health outcomes.

Organizational support has been proven essential to conduct adequate teamwork [34] and previous research has indicated that defining unit outcomes, as well as rewarding all members accordingly, may help engage all members in the process and enhance interdisciplinary collaborations [35].

Furthermore, adding the use of preventive medicine and health education to the clinics’ routine assessments may affirm the organizational support of such tools and help enhance its implementation. All these may help encourage all team members to make better use of preventive medicine and health education. Until such a time, as important as personal characteristics, training, or type of primary care models are, they may not sufficiently motivate health professionals to engage in preventive medicine more extensively.

This study had a few limitations.

The response rate was not high. Physicians’ under-representation may present a partial picture regarding preventative medicine implementation in their clinics. Since responding physicians represented all of MHS’ physicians with respect to gender and clinic size (with the exception of under-representation of small clinics, which is less relevant, as discussed in the methods section) we feel this bias did not profoundly flaw our study’s conclusions.

In addition, assessing rates of preventive medicine and health education tools implementation based on self-report could be biased by employees’ tendency to over-report activities preformed due to the need to better fit their own professional perception, or to meet MHS’s expectations. The automated questionnaire system was used to minimize this aspect as much as possible and further validation was gained through the CHR data, found to support our findings.

The Collaboration Model’s sample size was vastly different from the other two models. This difference is representative of MHS clinics and does not impair statistical conclusions deduced in this study.

## Conclusions

This study has provided new insights regarding variables affecting implementation of preventive medicine and health education tools in primary care. We found multidisciplinary models to be associated with higher levels of these tools’ implementation. While these results were based on health professionals self-report, it was also strongly supported by objective organizational computerized data.

Although the organizational approach to the Collaboration Model resembles that of the Independent Physicians, in terms of preventive medicine application, this model resembled the Teamwork model. This indicates that multidisciplinary support may help promote higher rates of preventive medicine and health education implementation as well as better patients’ health behaviors.

Supporting professionals’ training as well as acquiring collaboration skills is essential and may help promote the implementation of the tools acquired.

Our findings may assist health organizations and policy-makers in modifying practice attributes to enhance preventive medicine and health education implementation in primary care.

Further examination of patients’ health outcomes in future studies, may ascertain the link between preventative medicine and health education implementation and patients’ clinical outcomes in the various models.

## Data Availability

The data that support the findings of this study are available from Maccabi Health Services’ department of Health Services Research, but restrictions apply to the availability of these data, which were used under license for the current study, and so are not publicly available. Data are however available from the authors upon reasonable request and subject to MHS’ permission.

## Abbreviations

CHR: Computerized Health Records
HMO: Healthcare Maintenance Organization
MHS: Maccabi Healthcare Services
OR: Odds Ratio
